# Chicago Medical Society

**Published:** 1888-02

**Authors:** 


### CHICAGO MEDICAL SOCIETY.

STATED MEETING, October 17, 1887.

The President, W. T. Belfield, M. D., in the chair.

Dr. E. Fletcher Ingals reported a case of

### RETROPHARYNGEAL ABSCESS.

The following is one of unusual interest on account of the deep fistulous tract which remains, and as an illustration of the necessity for prompt incision for the evacuation of pus in cases of retropharyngeal abscess:

Mrs. H. P., æt. 44, was sent to me during the month of August from a neighboring State for an opinion. She complained of an oppressive purulent discharge from the pharynx, with more or less pain between the shoulders, cough and night-sweats. She stated that the discharge would occur every few days, and that afterwards she would be free from cough, pain or other annoyance for sometime, when the symptoms would again come on gradually and increase steadily until another evacuation of the pus occurred.

Six months before coming to see me she had been attacked by soreness and swelling in the naso-pharynx. The swelling had gradually extended downward, causing more or less difficulty in swallowing and respiration, and had been allowed to take its course for two months, when an opening was made in the lower part of the pharynx which gave vent to a large quantity of offensive pus. Subsequently the incision closed, was re-opened twice, and finally it had remained patent. While the swelling was still in the naso-pharynx she had for sometime also a purulent discharge from the right ear. I found the patient anæmic, weak and emaciated, weight only 94 pounds, instead of 112 pounds when in health. Pulse 132. Tongue red and glazed in centre, but the appetite good. The cough was not constant, but it usually troubled her considerably every day. The pains of which she complained were not severe, and were confined to the chest and shoulders.

Upon examining the chest I found no abnormal signs excepting slight feebleness of the respiratory sounds over the lower third of the right lung. A mucus polypus half closed the right naris; larynx normal. When the tongue was forcibly depressed I could see, a little to the right of the median line, at the lower third of the larynx, a small, red fungoid mass about the size of a pea. The vault of the pharynx was normal, and nothing else of an abnormal character could be seen. I removed the fungoid mass with a snare, and beneath it found an opening about two millimetres in diameter, into which I passed a small elastic catheter, which I gently pushed down the fistulous tract thirty centimetres, when she complained of its extremity causing her pain a little to the right of and below the right breast.

The position of the lower end of the fistula seemed in some way to account for the enfeebled respiration at the lower third of the right lung. The patient did not wish to remain in the city; I therefore was unable to see her when there was pus in the cavity, and consequently could not determine the exact position of the lower end of the fistula.

I recommended removal of the polypus and washing of the fistulous tract daily with carbolized water or peroxide of hydrogen through the catheter introduced to its bottom. Tonics were also suggested. The result of the examination was communicated to her physician, but I have heard nothing further of the case. I was under the impression that when the lower part of the sac was filled with pus it might be possible to detect it and make a counter-opening through the pleural sac whereby constant drainage could be established so that healing of the fistula might be effected.

Dr. F. O. Stockton said: It seems to me that this sequel of a retropharyngeal abscess is something a little out of the ordinary run. I must say that in my experience I have never met with anything that has produced any such symptoms, although I have frequently met with fistulas resulting from a retropharyngeal abscess. The case is interesting as showing what may occur when an abscess of this kind is not recognized in time. Abscesses are liable to burrow deeply, and in a location like this, where the soft tissues overlie a hard, bony substance, an abscess with a tendency to burrow could easily go very deep, and by pressing upon the nerves produce these pains, but it seems strange, unless the nerves were bare, that passing a small catheter down should produce these pains below the right breast. It looks as though some of the spinal nerves might have been laid bare by this burrowing of the pus. In a case of that kind I think what the doctor suggested was about the only thing that could be done, although I can hardly see how the opening he suggested could be made so as to give thorough drainage without injury to some nerve or other important structure.

Dr. W. E. Casselberry said that the subject is one of interest for two reasons: *First*, retropharyngeal abscess is not a common disease, especially in the adult. It is oftentimes overlooked, and the responsibility rests upon the physician in all cases where there is dyspnœa, continuous or paroxysmal, to make a thorough examination of the throat, both by inspection and palpation, in order to determine the diagnosis. *Secondly*, it is of interest on account of the peculiar complication in this case. I have seen the record of a similar case reported by Cohen among a number of cases of retropharyngeal abscess having some peculiarities. In this one a sinus burrowed deeply between the œsophagus and trachea and finally pointed externally low down in the neck, and pus was evacuated by incision. On account of the loose connective tissue in this region and the influence of gravity, the natural tendency is for the pus to burrow downward, and other cases of the sort will doubtless be observed.

In reply to Dr. Strong as to how he would make the opening suggested, Dr. Ingals said: I thought that when that sinus was filled with pus there might be a considerable collection, a pocket perhaps of a couple of inches in diameter, and if it was of that size I could find the location, and when found I believe I could get into it. I should not hesitate to plunge an aspirator needle into it, even passing it through the lung if necessary. I am reminded of a case in an infant that I saw so many years ago that I am not ashamed to tell of it. I then thought the child died of convulsions, but I think now that it died of retropharyngeal abscess. I know from reading of these cases that in young children sudden stopping of the breath occurs that seems as if from closure of the glottis, but there is none of the sound that occurs with spasm of the vocal cords. Last week I had a man of perhaps fifty years of age sent to me. The word that came with him was that he had stenosis of the larnyx, and that something must be done. I took my laryngeal mirror to examine his larnyx, and found a large swelling in the pharnyx, which I could see by depressing the tongue. It was a retropharyngeal abscess, which had existed four weeks. He swallowed with difficulty, but opening of the abscess relieved the dysphagia.

Dr. A. B. Strong read a paper on

### SUBPERIOSTEAL RESECTION OF A RIB FOR EMPYEMA IN CHILDREN—RECOVERY.

Dr. E. Fletcher Ingals: Some four years ago I reported fourteen cases of empyema I had treated, and since that time I have treated a number of others. I have had no experience with the method adopted by the reader of the paper, but one of my patients unfortunately had the operation done upon him. I have not very much sympathy with the operation, excepting as a last resort to allow of greater contraction of the chest. The author of the paper performed the operation skillfully and his patients recovered; I fear if I had performed it my patient would have died. But I can open the chest otherwise with safety to the patient and satisfaction to myself. I have treated several cases of empyema similar to those just reported, one very young child, perhaps a year and a half old, where recovery followed simple aspiration. I recall two others, which were similar to the first case reported, in which the disease had run from three to five weeks. Drainage tubes were inserted through a trocar, and the cavity washed out frequently, and in both the drainage tubes were required only between one and two weeks, and the patients were well within three weeks. I think they recover more speedily than they would have done if the rib had been resected. Another case I have in mind had been sick longer, two or three months, when I first saw it, and it was perhaps six weeks before the child recovered. In all of these cases the expansion of the lungs and chest wall became perfect.

I think children are quite as likely to recover by this method as when ribs are resected, and I think we avoid a great danger. I feel confident that I have seen cases die from free opening in the chest cavity, and I do not think it a wise practice to open the pleural sac in this manner. However, I have seen two cases die within a few hours after simple aspiration, but I do not know whether the removal of the pus was the cause of death or not. One was a child who had been suffering from scarlatina and was so nearly dead that she did not feel the puncture of the needle, and the other was an aged man. In the case reported of the boy 17 years of age, I think drainage tubes would have acted quite as well introduced through a trocar, though not quite as large tubes could have been introduced, but I do not think such a large tube necessary. With two drainage tubes one-eighth of an inch in their inside diameter, I think there is no difficulty in washing out the chest and doing it thoroughly.

One point spoken of in the paper I am glad came up, as it brings to mind something important in the operation: that is do not operate too low. In this case the 9th rib was resected; if it had been the 6th or 7th rib there would not have occurred the trouble which was experienced afterwards. I have had the same trouble through the introduction of drainage tubes too low. One patient, in whom the tube was introduced in the 7th or 8th interspace near the angle of the rib, wore it several months, but finally died of pneumonia of the opposite lung, and on examination I found the drainage tube for two inches in its course upward bound to the chest wall by the diaphragm which had crowded upward. My rule is to make the opening within an inch and a half of the surface of the fluid when the pleural sac is about half full; if the cavity was full I should go between the 6th and 7th ribs, or the 7th and 8th near the angle. The method that I have used in introducing drainage tubes would perhaps be of some interest. At present I use a broad flat trocar that is wide enough to allow the introduction of two of these tubes at once, that is two tubes with an inside diameter of one-eighth inch. First making a very small opening in the skin with a scalpel, the trocar is then plunged in the cavity, and as the stiletto is withdrawn, I place my thumb over the canula to stop the flow of pus; the tubes that have been prepared beforehand are then slipped rapidly through the canula to the required depth, and the canula is at once withdrawn. No air will have entered the chest and not more than two ounces of pus will have escaped. To make the tubes I take a piece of two feet in length, cut it half way across near the middle, fold it on itself and stitch the two sides together so that the tubes can be spread apart in the chest. In one of these tubes I make openings for about four inches, in the other one or two openings close to its extremity. The outer ends of the tubes are tied tightly so that the pus will not flow till I am ready to fasten the tubes in. I cut off two half-inch sections from the same tubing and pass through each two bits of string, the ends of which are tied in loops; these are carried over the drainage tubes down to the surface of the chest; long strips of adhesive plaster are then passed through the loop and fastened to the chest when the tubes will be firmly fixed. I then carry a rolled bandage around the chest over the strips and the dressing is complete. If the patient is in good condition, I then attach to each drainage tube, by means of a bit of glass tubing, longer sections of tubing, which have been filled with water, that can reach to the bottom of a basin of water sitting on the floor; the strings which have fastened the ends of the drainage tube are then cut and the pus allowed to escape, or such part of it as is deemed advisable. I recall one case in which I am satisfied if I had allowed any quantity of pus to escape at first it would have been fatal. On one or two occasions I did allow more to escape than was desirable, and I had to inject water into the chest to replace it.

The after-treatment that I have employed consists mainly in washing out the chest twice a day with a solution of carbolic acid 2 per cent. at 100 F. With tubes placed in this way the patient's friends can perform this office, and the physician will not be obliged to see the patient but a few times, once a week or once in two or three days, according to the intelligence of the attendants. I think, however, that in adults, where the lung expands with more difficulty, one should be very careful to watch the case about the fifth or sixth week to insure final closure of the cavity. I have seen cases in which at this time I think the closing up of the cavity ceased from lack of proper stimulation. It is very easy to measure the size of the cavity by filling it up with water and measuring the water as it flows out. At first the cavity will rapidly diminish in size, but after two or three weeks it closes more slowly, and after the fifth or sixth week the healing will cease in some cases. At this time I have used satisfactorily washes of solution of the sulphate of zinc or copper; I like best the sulphate of zinc. In two cases of adults, where I could not watch them closely at this time, I have had some difficulty later on in closing the cavity. I stated in the beginning that I had personal experience in only one case in which resection of the rib had been done. The patient had been sick about two years when I first saw him. I introduced the two drainage tubes as prescribed, and the cavity closed until it would only hold about an ounce; but there seemed no way to get this cavity to heal; however, the gentleman was satisfied and went about his business, until finally he fell and broke his arm and went into the hospital. While there it was concluded to resect one of the ribs so as to allow the cavity to close entirely. In resecting the rib an artery was cut, and the surgeon had great difficulty in stopping the hemorrhage. The resection did no good, though larger drainage tubes were introduced; they did not cleanse the cavity better than the smaller ones, and the man still wears them. From my experience I believe that where two tubes are introduced, as I have directed, and washing is carried on through first one and then the other, there is no difficulty in cleansing the cavity perfectly, unless gangrene has occurred. Flakes of lymph will be readily washed out, or if too large they will be disinfected so as to remain harmless until they finally break down or become organized with the new tissue that binds the two pleural surfaces firmly together.

Dr. John D. Skeer: I have not had much experience in exsection of the ribs for drainage purposes, but as far as the operation is concerned it is certainly not a very formidable one when performed, as Dr. Strong has described it, by making the operation subperiosteal. I have had a great deal of trouble in my practice in the drainage of chest cavities with small drainage tubes. I recall a number of cases in the army, and I think now that if I had those cases under my care I would exsect a portion of the rib. The rapid flow of matter under these circumstances does not necessarily follow. The opening into the cavity may be small or large, according to the size of the drainage tubes, and the flow of matter can be moderated according to the requirements of each individual case. The importance of this grows out of the fact that the lung becomes bound down by bands and adhesions which prevent its rapid expansion, and there may possibly be ulcerated spots admitting air into the pleural cavity, and pus into the lung tissues, from a too ready expansion of the lung, and it takes time for these bands and adhesions to soften down, relax and expand to accommodate the lung in its natural expansion. We should differentiate between *serous effusion* into the pleural cavity and suppurative inflammation of the pleura.

Dr. H. T. Patrick: My experience with this operation of resection is limited to one case, which I had under observation three years ago. It was not a case of simple empyema, but a pneumopyothorax of a boy ten years of age, of over a year's standing, with considerable retraction of the chest and a small external opening. Three ribs were resected, the 6th, 7th and 8th. The ribs were markedly overlapping one another, and for that reason more were removed. The boy made a very satisfactory and rapid recovery; he had considerable temperature and was much emaciated, but immediately after the operation, the chest being washed out once a day, he lost his temperature and at once began to flesh up, and in a few weeks was entirely well. In regard to the operation, my idea is that it should be limited to this class of cases.

I should say that in empyema aspiration, incision and drainage, and resection of the rib, should be the order of procedure. In Dr. Strong's second case the lung failing to expand well points out one of the weak points of the operation as a primary procedure. Had aspiration been performed first, I think the conditions would have been more favorable for expansion than by making as free an opening as for resection and allowing air to enter the cavity. I think we follow right in the line of nature's efforts in these cases of retraction of the chest wall; we allow the chest to fall in and approximate itself to the other surface. There are other dangers connected with this operation as a primary procedure: one is that there are cases on record where, under an anæsthetic, the cavity has ruptured into a bronchus and the patient has choked to death. Deaths have also been recorded from simply washing out the cavity.

Dr. N. H. Pierce: There is one thing that I would like to suggest in this connection, and it is this: As I understand it, we resect a rib to simply enlarge our area of drainage. In a case that I had in the last month, where I had drawn off nearly three gallons of fluid in three *tempos*, I first merely inserted a drainage tube one-half inch in diameter; this drain was constantly being plugged up by coagula, or whatever it might have been, thus causing retention and absorption of the purulent matter, followed by the usual symptoms. I resected a rib after that and introduced a drainage tube one inch in diameter, with the advantage of not having it clogged up again; the patient is making a good recovery. Another point is that there have been a number of cases reported from the Berlin clinic of death from drowning occurring from washing out the cavity while the patient was under ether, there being a communication between the pleural cavity and the lungs. It is well, therefore, that the primary cleansing be delayed until the day following the operation. I have, in three cases of this character, let out the entire contents of the cavity, amounting in each case to three or four pints, with no abnormal result, using as an antiseptic lavage a saturated solution of boracic acid. There have been deaths reported from the use in the pleural cavity of carbolic acid, thymol and salycilic acid, and the only agent that has not been proven more or less dangerous when used in the pleural cavity is boracic acid, and Dr. Fraenkle insists that this is the only thing that should be used in this capacity.

Dr. A. B. Strong was very glad to find that his article had excited so much interest. It is certainly a practical subject, and one we are likely to meet with any day, and I am yet in favor of resection. It is my impression that the first resection was made about fifteen years ago. A point against resection was raised by Dr. Ingals—that of the too rapid evacuation of the matter. That point was ably answered by Dr. Skeer, who said that when we have free evacuation we have a corresponding influx of air which takes the place of pus. I believe that to be so. In the two cases reported there was considerable cough at the time the chest was opened, but that quickly subsided. I had no fear that there would be rupture of the lung; I could feel the pleura three-fourths of an inch thick. Until some better reasons are given for not operating, I shall certainly be in favor of making a free opening by resection. I want to make an opening large enough so that I can get into the cavity and evacuate it freely; I cannot get out enough pus through an eighth of an inch opening to satisfy me. In the case of the older boy, shreds of lymph as large as the little finger were thrown out for several days. I have no fear in opening the chest freely and draining it freely.

